# Breast cancer brain metastases: the last frontier

**DOI:** 10.1186/s40164-015-0028-8

**Published:** 2015-11-24

**Authors:** José Pablo Leone, Bernardo Amadeo Leone

**Affiliations:** University of Iowa Holden Comprehensive Cancer Center, University of Iowa Hospitals and Clinics, C32 GH, 200 Hawkins Drive, Iowa City, IA 52242 USA; Grupo Oncológico Cooperativo del Sur (GOCS), Rivadavia 360, 8300 Neuquén, Argentina

**Keywords:** Breast cancer, Brain metastasis, Metastatic breast cancer

## Abstract

Breast cancer is a common cause of brain metastases, with metastases occurring in at least 10–16 % of patients. Longer survival of patients with metastatic breast cancer and the use of better imaging techniques are associated with an increased incidence of brain metastases. Unfortunately, patients who develop brain metastases tend to have poor prognosis with short overall survival. In addition, brain metastases are a major cause of morbidity, associated with progressive neurologic deficits that result in a reduced quality of life. Tumor subtypes play a key role in prognosis and treatment selection. Current therapies include surgery, whole-brain radiation therapy, stereotactic radiosurgery, chemotherapy and targeted therapies. However, the timing and appropriate use of these therapies is controversial and careful patient selection by using available prognostic tools is extremely important. This review will focus on current treatment options, novel therapies, future approaches and ongoing clinical trials for patients with breast cancer brain metastases.

## Background

Breast cancer represents the second most frequent cause of brain metastases after lung cancer, with metastases occurring in 10–16 % of patients [1]. In addition, autopsy studies have demonstrated another 10 % which were asymptomatic [2]. The incidence of brain metastases seem to have increased in recent years, this is likely due to prolonged survival of patients receiving more efficient treatments and the availability of better imaging techniques that lead to increased detection of brain metastases.

The development of brain metastases is a complex process, requiring invasion of the primary breast cancer cells into surrounding tissue and vessels, traffic through the circulatory system and colonization and growth in the brain parenchyma [3, 4]. In breast cancer, this process takes a median of 32 months from the initial cancer diagnosis [5]; which shows that the breast cancer tumor cells, unlike other cancer cells, need more time to develop the ability to penetrate through the blood–brain barrier (BBB) and colonize the brain. There is also a selective pressure that can make the brain a preferential site of metastasis, as many of our currently available therapies are unable to cross the BBB, even if this barrier is disrupted by tumor invasion.

Previous studies have identified the subgroups of patients with triple-negative and human epidermal growth factor receptor 2 (HER2)-positive breast cancer as having an increased risk for the development of brain metastases [6–9], with up to half of patients with HER2-positive metastatic breast cancer experiencing brain metastases over time [10]. Tumor subtypes are also an important factor for the median time interval from primary diagnosis to development of brain metastases; a recent large study showed shorter intervals for triple-negative and HER2-positive patients, and longer intervals for estrogen receptor (ER) positive tumors [11].

Brain metastases in breast cancer patients represent a catastrophic event that portends a poor prognosis, with a median survival that ranges from 2 to 25.3 months despite treatment [5, 12–14]. In addition, brain metastases are a major cause of morbidity, associated with progressive neurologic deficits that result in a reduced quality of life [15]. With the advent of better systemic therapies, brain metastases constitute an increasing clinical problem. This is particularly important in HER2-positive patients, in whom brain metastases can occur in the setting of controlled extracranial disease [16]. In contrast, it is common for patients with triple-negative breast cancer to develop brain metastases with concurrent extracranial disease progression [17]. Treatment options for patients with breast cancer brain metastases are limited and include surgical resection, whole-brain radiation therapy (WBRT), stereotactic radiosurgery (SRS), chemotherapy and targeted therapy [12, 18, 19]. This review will focus on the key issues of current treatment options, comment on novel therapies and ongoing clinical trials for patients with breast cancer brain metastases.

## Prognostic factors

The prognosis of patients with breast cancer who develop brain metastases is affected by several factors. Tumor subtypes have been identified as a prognostic factor for overall survival in brain metastases [20, 21]. Triple-negative breast cancer patients have the shortest survival ranging from 3 to 4 months [9, 16, 22]. In contrast, patients with HER2-positive tumors have longer survival than those with triple-negative or luminal subtypes, although their rates of brain metastases are higher [9, 16, 23].

Another important prognostic factor is the performance status of the patient at the time of diagnosis of brain metastases. Most studies have established the utility of the Karnofsky Performance Status (KPS) as a tool to assess prognosis and identified that patients with longer survival have KPS scores ≥70 [13, 14, 24]. In addition to the KPS, patient’s age can also affect prognosis. Older age at the time of initial breast cancer diagnosis has been associated with shorter overall survival and shorter survival from the time of first tumor relapse [5, 25]. Finally, the burden of disease represented by the number of brain metastases, as well as the presence of uncontrolled extracranial disease have both been related with worse prognosis [23, 24, 26].

One of the most frequently used tools for the assessment of prognosis in brain metastases is the graded prognostic assessment (GPA) [27]. This prognostic index includes age, KPS score, number of brain metastases and extracranial metastases. After its original validation [28], the index was modified to create a breast cancer-specific GPA that included tumor subtypes among its prognostic factors [11, 14]. However, number of brain metastases was not incorporated into the final model. A recent study validated the breast cancer-specific GPA and refined it with the addition of number of brain metastases [29]. This represents a very useful tool for patient risk assessment and selection for clinical trials.

## Local therapy modalities

### Surgical resection

Surgical resection of the brain metastasis is an important treatment option in patients with single or few (≤3) lesions. Particularly when the systemic disease is well controlled and when the brain metastases are symptomatic. Although the anatomic location of the metastatic lesion can be a limitation, surgical resection has additional advantages including the potential for immediate improvement of focal deficits, relief of intracranial hypertension and establishment of histological diagnosis in patients with no other site of metastasis.

One of the first studies to evaluate the role of surgical resection in brain metastasis was conducted by Patchell et al. [30]. In this study, 48 patients with single brain metastasis from any primary were randomized to either surgical resection of the brain metastasis followed by WBRT or needle biopsy followed by WBRT. Brain recurrence was less frequent in the surgery group compared with the radiation group (20 vs. 52 %, respectively; *P* < 0.02). Median overall survival was longer in the surgery group (40 weeks) compared with the radiation group (15 weeks) (*P* < 0.01). Neurological outcomes were also improved with surgery where patients remained functionally independent longer (median, 38 vs. 8 weeks in the radiation group; *P* < 0.005).

A subsequent study randomized 63 patients with systemic cancer and a single brain metastasis to surgical resection plus WBRT vs. WBRT alone. The combined modality led to longer overall survival and longer functionally independent survival compared with WBRT alone, particularly in patients with stable extracranial disease (median overall survival 12 vs. 7 months, respectively; median functionally independent survival 9 vs. 4 months, respectively) [31]. Patients with progressive extracranial disease had similar outcomes irrespective of treatment, a finding that was also observed in another randomized trial [32]. Three non-randomized studies have also confirmed improvements in survival, brain recurrence and neurological outcomes with surgical resection in addition to WBRT [33–35].

### Stereotactic radiosurgery

In patients with limited brain metastases who are deemed poor candidates for surgical resection or who have lesions in difficult anatomic locations, SRS has been proposed as an alternative treatment option. This approach delivers a high-precision photon radiation to a small target volume while sparing most normal brain tissues. SRS has an advantage over WBRT in that it avoids the feared toxicity of neurocognitive decline that is associated with the latter intervention [36–38].

The efficacy of SRS for local control of brain metastases has been demonstrated in a study conducted by Kondziolka et al. [36], were median time to local failure in patients with two to four brain metastases was significantly improved from 6 months with WBRT alone to 36 months with the addition of SRS (*P* = 0.0005). In spite of this improvement in local control, overall survival was unchanged and was related to the extent of extracranial disease. The Radiation Therapy Oncology Group (RTOG) confirmed the efficacy of SRS in addition to WBRT in patients with one to three brain metastases and documented an overall survival improvement of 1.6 months in the subgroup of patients with single unresectable brain metastasis who received the combined therapy [39]. In patients with solitary metastases who are treated with surgical resection plus WBRT, the addition of SRS to the tumor bed can improve local control [40].

Subsequent randomized studies that included brain metastases from different cancers demonstrated that patients with one to four lesions who were treated with SRS alone had similar survival and improved neurocognition compared with patients who received both SRS and WBRT, however local control was inferior with SRS alone [41, 42]. These results were confirmed in a meta-analysis [43]. A recent non-randomized non-inferiority trial showed the efficacy of SRS without WBRT for overall survival in patients with five to ten brain metastases to be not inferior to the same treatment in patients with two to four lesions [44]. Despite the findings of this study and others showing similar clinical outcomes [45–47], currently there is no randomized data to support the use of SRS without WBRT in the treatment of brain metastases for patients with >4 lesions.

### Whole-brain radiation therapy

One of the most important treatments available for brain metastases is WBRT, particularly in the setting of multiple brain lesions. This approach has two main goals—the control of macroscopic metastases, and the eradication of microscopic seeding of the brain. The majority of patients are given conventional WBRT, a total dose of 30 Gy in 10 fractions with daily fractions of 3–4 Gy [48].

The benefit of WBRT after surgical resection has been demonstrated in a prospective trial that randomized 95 patients who had single brain metastases to WBRT or observation [49]. The study showed that patients in the WBRT group had fewer recurrences both at the operative site (10 vs. 46 %, *P* < 0.001) and at other sites in the brain (14 vs. 37 %, *P* < 0.01), however overall survival was not increased.

Substantial controversy exists about the role of WBRT in patients with few (≤4) brain metastases. In this setting, treatment with WBRT after surgical resection or SRS resulted in fewer intracranial recurrences, but there was no difference in overall survival [41, 50]. Associated toxicities with WBRT included worse neurocognitive outcomes and quality of life [42, 51]. However, withholding WBRT can lead to progressive disease in the brain, which in turn could also negatively impact cognition [52, 53]. This issue was addressed in a study conducted by the North Central Cancer Treatment Group (NCCTG) N0574 where patients with one to three brain metastases were randomized to SRS or SRS plus WBRT, the study showed more frequent decline in cognitive function with the addition of WBRT despite better brain control [54]. Therefore, delaying or avoiding the administration of WBRT in metastatic breast cancer after surgical resection or SRS through the careful use of effective systemic therapies, could provide substantial benefits in terms of quality of life, particularly in patients with high GPA scores in whom survival is expected to be longer [14, 29]. This approach results even more appealing when one considers that none of the above mentioned randomized trials have shown overall survival gain with the addition of WBRT.

## Systemic therapies

The mainstay of systemic treatment for breast cancer brain metastases is cytotoxic chemotherapy; however, there are currently additional options for targeted therapy. Therefore, it is crucial to consider the tumor subtype not only for prognosis but also to understand the different options for systemic therapies.

### Hormone receptor-positive

Patients with ER-positive brain metastases derive substantial benefit from systemic chemotherapy. Niwinska et al. reported improvements in median survival from 3 to 14 months with the addition of systemic therapy in patients with luminal breast cancer [20]. A similar result was seen in another study where the median overall survival of patients with luminal breast cancer was improved from 7.1 to 14.3 months with chemotherapy [55].

The efficacy of endocrine therapy for brain metastases is less clear, since most randomized trials testing this intervention excluded these patients. Despite of this, there are some reports showing response of brain metastases to tamoxifen, megestrol acetate and aromatase inhibitors [56–61]. Interestingly, tamoxifen and its metabolites can achieve high concentrations in the brain. Lien et al. showed that the concentrations were up to 46-fold higher in the brain metastatic tumor and brain tissue than in serum [62]. Taken together, these data support the hypothesis that in patients with metastatic ER-positive breast cancer who have asymptomatic systemic disease and locally treated brain metastases treatment with endocrine therapy could be considered prior to systemic chemotherapy. Clinical trials evaluating this approach should be conducted.

### HER2-positive

Patients with HER2-positive metastatic breast cancer have experienced a dramatic improvement in overall survival with optimal the utilization of HER2 targeted therapy [63]. Unfortunately, the advances in systemic treatments for these patients came in hand with an increase in the rate of brain metastases, which now poses a significant threat [10]. The efficacy of anti-HER2 therapy to control systemic disease for longer periods of time has exposed the ability of the HER2-positive breast cancer cells to seed the brain parenchyma and develop brain metastases. Most chemotherapy agents and HER2 targeted therapies do not cross the intact BBB or are pumped out of the central nervous system (CNS) by P-glycoproteins present in the BBB, therefore they may not reach sufficient therapeutic levels to eradicate metastatic cells [1]. For example, in patients without brain metastases, the ratio of trastuzumab in plasma to trastuzumab in cerebrospinal fluid is >300:1 [64, 65]. The brain then, can serve as a sanctuary where those cells that have the ability to seed can escape the cytotoxic efficacy of systemic therapy. However, tumor growth in the brain as well as cranial surgery and brain radiotherapy can disrupt the BBB and allow access of systemic drugs to the tumor. This concept has been proven by a number of labeled-trastuzumab imaging studies [66, 67]. Also, several clinical studies have shown that the combination of chemotherapy with trastuzumab improved survival, even after the development of brain metastases [68–70]. This benefit is presumed to be mainly due to improved control of systemic disease [71].

Lapatinib, a small molecule with potential ability to cross the BBB, has been extensively tested in the treatment of HER2-positive brain metastases. As a single agent, lapatinib has shown response rates in the brain ranging from 2.6 to 6 % in heavily pre-treated patients [72, 73]. However, when added to capecitabine, response rates increase to 20 to 33 % [73–77]. The highest efficacy is observed in previously untreated patients, where the combination of lapatinib and capecitabine produces an objective response rate of 65.9 %, with a median time to progression of 5.5 months and a 1-year survival rate >70 % [78]. This drug combination has also shown to reduce the rate of brain metastases as the first site of progression from 6 % with capecitabine alone to 2 % with capecitabine and trastuzumab (*P* = 0.045) [79]. The efficacy of lapatinib to prevent brain metastases was further tested in the CEREBEL trial, where patients with HER2-positive metastatic breast cancer without CNS metastases were randomized to lapatinib or trastuzumab in combination with capecitabine. The primary end point of the study was incidence of CNS metastases as first site of relapse. The study was terminated early and showed no difference between arms for the incidence of CNS metastases (3 % for lapatinib vs. 5 % for trastuzumab, *P* = 0.36), however progression-free survival and overall survival were longer with trastuzumab and capecitabine [80]. Despite the low incidence of CNS metastases seen during the study, it is important to notice that 4.7 % of all screened patients were excluded due to detection of asymptomatic brain metastases.

In the EMILIA trial, Trastuzumab emtansine (T-DM1), a novel antibody–drug conjugate, improved overall survival compared with lapatinib plus capecitabine in patients with previously treated HER2-positive metastatic breast cancer [81]. A recent retrospective, exploratory analysis of this trial focusing on patients with baseline CNS metastases, showed that the rate of CNS progression was similar for both arms, however median overall survival in patients with CNS metastases at baseline was significantly improved with T-DM1 (26.8 vs. 12.9 months, *P* = 0.008) [82]. Similar results were seen in the CLEOPATRA trial, where patients with HER2-positive first line metastatic breast cancer experienced significant improvements in progression-free and overall survival with pertuzumab, trastuzumab and docetaxel compared with placebo, trastuzumab and docetaxel [63]. In this trial, an exploratory analysis of the incidence and time to development of CNS metastases as first site of disease progression, also showed that the incidence was similar between the two arms, however the time to development of CNS metastases was significantly prolonged in the pertuzumab arm from 11.9 to 15 months (*P* = 0.0049) [83]. Taken together, the data from EMILIA and CLEOPATRA underscore the importance of systemic disease control for improving overall survival in patients with brain metastases.

Given the high prevalence and impact that brain metastases cause in patients with HER2-positive breast cancer, the American Society of Clinical Oncology (ASCO) published in 2014 its first clinical practice guideline on the management of patients with HER2-positive brain metastases [84]. Some of the key recommendations included the following: (a) for patients with progressive intracranial metastases, options include a trial of systemic therapy in addition to other local therapy modalities; (b) for patients whose systemic disease is not progressive at the time of brain metastasis diagnosis, systemic therapy should not be changed; (c) for patients whose systemic disease is progressive at the time of brain metastasis diagnosis, treatment should include HER2-targeted therapy according to the algorithms for treatment of HER2-positive metastatic breast cancer [85].

### Triple-negative

Patients with brain metastases from triple-negative breast cancer unfortunately lack targeted therapies and chemotherapy is currently their only systemic option.

Some of the initial studies of patients with brain metastases have shown objective response rates of around 50 % with traditional chemotherapy combinations [86, 87]. A study evaluating cisplatin with etoposide showed 38 % response rate in the brain [88]. Topotecan and temozolamide have failed to show responses as single agent [89, 90]. However, when temozolamide was combined with cisplatin had 40 % response rate [91], and showed 18 % response rate when combined with capecitabine [92]. The experience with single agent capecitabine is limited to mostly retrospective studies [93].

It is important to keep in mind that while different chemotherapies will defer in their ability to penetrate the BBB, most brain metastases will significantly disrupt this barrier. Therefore the ability to deliver systemic chemotherapy to the brain metastasis is not much different from the ability to deliver chemotherapy to that tumor anywhere else in the body. Hence, treatment efficacy is more closely related to tumor chemosensitivity than to the drug ability to cross an intact BBB and this hypothesis has been proven in several of the above mentioned studies [86–88]. Brain metastases tend to be chemotherapy-resistant because they tend to occur late in the natural history of breast cancer and by that point many times the breast cancer is already chemotherapy-resistant.

## Suggested treatment approach

Based on the evidence reviewed above, we suggest the following management approach:

- For patients with a single brain metastasis, surgical resection can improve overall survival, particularly in symptomatic patients when systemic disease is well controlled. The addition of SRS to the tumor bed or WBRT improve local control.
- For patients with one to four brain metastases, SRS with or without WBRT should be considered to improve local control. If WBRT is added, we recommend to delay its administration as much as possible to prevent neurocognitive decline, which is particularly important in the absence of overall survival benefit. Surgery can be considered for large or symptomatic lesions.
- For patients with more than four brain metastases, WBRT can be the treatment of choice to palliate symptoms and improve local control. There are no randomized trials to support the use of SRS in this setting.
- For patients with progressive systemic disease at the time of development of brain metastases, a change in systemic therapy should be considered based on the tumor subtype.
- For patients with non-progressive systemic disease at the time of development of brain metastases, systemic therapy should not be changed.
- For each patient, the choice of systemic therapy should be considered based on the tumor subtype.
- For patients with poor prognosis, options include WBRT and/or best supportive care.

## Novel approaches and future directions

Given the paucity of effective treatment options for patients with breast cancer brain metastases, this currently represents an area of great potential for future research. The use of bevacizumab has shown good results in patients with glioblastoma and has made the strategy of blocking the vascular endothelial growth factor (VEGF) pathway an interesting alternative to treat brain metastases. Two studies evaluating this approach have been reported. One showed CNS response rate of 63 % for bevacizumab and carboplatin; and the other one showed a response rate of 60 % for bevacizumab, etoposide and cisplatin [94, 95]. It is important to consider that VEGF blockade rises a number of controversies. One of the most concerning being that meta-analyses have failed to show an overall survival benefit with the use of bevacizumab in metastatic breast cancer, which resulted in the US Food and Drug Administration (FDA) withdrawal of the conditional approval of the drug.

The high affinity folate receptor (HFR) is a novel target present in 33 % of breast cancers for which there are available drugs being evaluated. Despite of the initial excitement, a recent study showed very low levels of expression in brain metastases [96]. Another approach that is currently under study in patients with breast cancer brain metastases is targeting the phosphatidylinositol 3-kinase (PI3K)—mammalian target of rapamycin (mTOR) pathway. This is one of the most commonly altered pathways not only in metastatic breast cancer but also in brain metastases [97]. Everolimus is being evaluated in combination with capecitabine and lapatinib (NCT01783756) and in combination with vinorelbine and trastuzumab (NCT01305941) in patients with HER2-positive brain metastases.

Given the high incidence of brain metastases, particularly for patients with HER2-positive and triple-negative breast cancer, there have been efforts to develop strategies for prevention of brain metastases in the high-risk subgroups. Much of these have evolved around the concept of using prophylactic cranial irradiation (PCI) in patients with these breast cancer subtypes, similar to what is currently done for patients with small cell lung cancer. However, as mentioned earlier in this article, the timing of development of brain metastases in patients with HER2-positive and triple-negative breast cancer is not the same, nor is the same their survival after development of metastatic disease. In addition, given the cognitive effects of brain radiotherapy, there is significant controversy around the optimal timing of PCI.

Table 1 shows a summary of currently ongoing studies evaluating different treatment strategies in breast cancer brain metastases.

**Table 1:** Ongoing clinical trials in breast cancer brain metastases

Tumor subtype	Treatment/target	Experimental arm	Control arm	Clinicaltrials.gov ID/phase
All	Chemotherapy	Cabazitaxel	None	NCT02166658 Phase II
		TPI 287	None	NCT01332630 Phase II
		ANG1005	None	NCT02048059 Phase II
		ANG1005 + trastuzumab (if HER2-positive)	None	NCT01480583 Phase II
		Liposomal cytarabine + high-dose methotrexate	None	NCT00992602 Phase II
	VEGF	Bevacizumab + carboplatin + trastuzumab (if HER2-positive)	None	NCT01004172 Phase II
		Bevacizumab + etoposide + cisplatin followed by WBRT	WBRT alone	NCT02185352 Phase II
		Sorafenib + WBRT	None	NCT01724606 Phase I
		Cabozantinib + trastuzumab (if HER2-positive)	None	NCT02260531 Phase II
	Radiation	WBRT + temozolamide	None	NCT02133677 Phase II
		WBRT with hippocampal avoidance	Conventional WBRT	NCT01942980 Phase III
		WBRT + efaproxiral + oxygen	WBRT + oxygen	NCT00083304 Phase III
HER2-positive	Chemotherapy	Cabazitaxel + lapatinib	None	NCT01934894 Phase II
	HER2	Lapatinib + WBRT	WBRT alone	NCT01622868 Phase II
		Neratinib ± capecitabine	None	NCT01494662 Phase II
		Afatinib ± vinorelbine	Investigator’s choice	NCT01441596 Phase II
		T-DM1 + WBRT	None	NCT02135159 Phase I
		ARRY-380 + trastuzumab	None	NCT01921335 Phase I
	mTOR	Everolimus + trastuzumab + vinorelbine	None	NCT01305941 Phase II
		Everolimus + lapatinib + capecitabine	None	NCT01783756 Phase Ib/II
	PI3K	BKM120 + trastuzumab ± capecitabine	None	NCT01132664 Phase Ib/II
	MET	Tesevatinib + trastuzumab	None	NCT02154529 Phase Ib/IIa
	Radiation	PCI	None	NCT00916877 Phase I
		PCI + taxane + trastuzumab	Taxane + trastuzumab	NCT00639366 Phase III
		SRS + HER2 directed therapy	None	NCT01924351 Phase II
Triple-negative	PI3K	BKM120 + capecitabine	None	NCT02000882 Phase II
	PARP	Iniparib + irinotecan	None	NCT01173497 Phase II
	Radiation	PCI	Observation	NCT02448576 Phase III
Hormone receptor-positive	CDK4/6	Abemaciclib	None	NCT02308020 Phase II

*CDK* cyclin-dependent kinase, *HER2* human epidermal growth factor receptor 2, *mTOR* mammalian target of rapamycin, *PARP* poly ADP ribose polymerase, *PCI* prophylactic cranial irradiation, *PI3K* phosphatidylinositol 3-kinase, *SRS* stereotactic radiosurgery, *VEGF* vascular endothelial growth factor, *WBRT* whole-brain radiation therapy

## Conclusions

Brain metastases are an increasing problem in breast cancer. They represent an unmet need for which more efficacious therapies are urgently required. A better understanding of the molecular underpinnings of CNS progression is needed and ongoing studies analyzing matched tissue from primary and brain metastases will hopefully shed light on this. Traditionally, patients with brain metastases were excluded from clinical trials evaluating systemic therapies and we are left with the unanswered question of how efficacious those therapies would be for patients with brain metastases. To this end, the large number of ongoing breast cancer-specific brain metastases trials is a step in the right direction.
